# Generation of high-uniformity and high-resolution Bessel beam arrays through all-dielectric metasurfaces

**DOI:** 10.1515/nanoph-2021-0603

**Published:** 2022-01-11

**Authors:** Lei Chen, Saima Kanwal, Binbin Yu, Jijun Feng, Chunxian Tao, Jing Wen, Dawei Zhang

**Affiliations:** Engineering Research Center of Optical Instrument and Systems, Ministry of Education and Shanghai Key Lab of Modern Optical System, University of Shanghai for Science and Technology, No. 516 Jun Gong Road, Shanghai 200093, China; Wenzhou Institute, University of Chinese Academy of Sciences, Wenzhou, Zhejiang 325000, China; Oujiang Laboratory, Wenzhou, Zhejiang 325000, China

**Keywords:** beam array, Bessel beam, high-uniformity, metasurface, non-diffractive beam, subwavelength

## Abstract

Bessel beam arrays are progressively attracting attention in recent years due to their remarkable non-diffracting nature and parallel manipulation capabilities in diverse applications. However, the poor phase discretization of conventional approaches such as spatial light modulators leads to low numerical aperture (NA) beam arrays due to the limitation imposed by the Nyquist sampling theorem and poor uniformity of the beam intensity. The key contribution of this study is to experimentally demonstrate the generation of high-uniformity and high-resolution Bessel beam arrays by utilizing all-dielectric metasurfaces. This is attained by optimizing the design of the supercell of a Dammann grating, particularly decreasing each supercell of the grating to a proper size. We demonstrate a 4 × 4 array of Bessel beams with a subwavelength transverse dimension (570 nm, ∼0.9*λ*) and a large NA of 0.4 for each beam in the array, while maintaining a relatively high uniformity intensity (52.40%) for the array. Additionally, the Bessel beam arrays are generated in a broadband range through the proposed all-dielectric metasurfaces. Our results are of great significance and particularly useful for applications of metasurface-based Bessel beam arrays in multidisciplinary fields such as laser fabrication, biomedical imaging, data storage, and multi-particle trapping.

## 1 Introduction

Beam array patterns serve as an ideal tool in numerous scientific fields and emerging applications, such as optical trapping, optical storage, laser fabrication, and microscopy, among others. They have attracted particular attention as a mature and versatile optical manipulation technique based on wavefront shaping for arrays of traps, at the user’s will [1, 2]. Traditionally, a beam array pattern is generated by a spatial light modulator (SLM)-based optical setup with controllable phases imposed on the SLM, which are conjugate to the back aperture of the objective [3], [4], [5], [6], [7], [8], [9]. However, mismatching of the accurate phase pattern at the back aperture of the high numerical aperture (NA) objective in the 4f system can deteriorate the quality of the beam array pattern [9]. Such focused Gaussian beam array patterns based on bulky objective lenses with limited working distances hinder the observation and manipulation of particles at greater penetration depths [2, 10]. Therefore, it is highly desirable to develop a miniature and cost-efficient device that can produce high-uniformity, high-resolution beam arrays with a long depth of focus for various practical applications.

Metasurfaces are an advanced class of ultra-thin optical elements composed of sub-wavelength nanostructure arrays [11], [12], [13], [14], [15], [16], [17], [18], [19], [20], [21], [22], [23], [24], [25], [26], [27], [28], [29], [30], [31], [32], [33], [34], [35], [36]. In recent years, their utilization has driven significant progress in holograms [18, 19], generation of non-diffractive beams [20], [21], [22], [23], [24], [25], [26], [27], [28], [29], [30], and beam array control [31], [32], [33], [34], [35], [36], among others. For instance, the generation of independently selective diffraction orders of beam patterns was demonstrated [32]. Moreover, three-dimensional (3D) optical vortex array patterns with spatially variant topological charge distribution based on a Dammann vortex grating and spiral zone plates were realized [33]. Elsewhere, vortex beam array patterns generated by metasurfaces with both phase and amplitude manipulation of the light were realized [34]. However, the aforementioned beam patterns are in the form of Gaussian focused fields with limited depths of focus, which prevent optical manipulations at deep penetration depths. Bessel beams, exhibiting radially symmetric non-diffracting transverse profiles [37] can be generated by dielectric metasurfaces to produce solid and hollow spots with high NA [21, 22]. Other important works have been reported for generating Bessel beams based on plasmonic metasurface [27], transmissive metasurfaces [28], catenary nanostructures [29], and dielectric meta-walls [30]. This provides a theoretical and experimental basis for the design of Bessel beam arrays with sub-wavelength transverse profiles and long depths of focus (i.e., high penetration depths for optical manipulation).

Recently, a multifocal metalens was proposed to generate Bessel beam arrays with a count of focal spots depending on the total number of nanopillars inside the unit cell of the metalens [36]. However, it is challenging to increase the number of channels of Bessel beams generated by the above method because supplementary nanostructures have to be placed inside the unit cell. To alleviate this difficulty, Dammann gratings can be used, which demonstrate great potential in the generation of beam arrays with a dense arrangement of focal spots [38]. This has led the researchers to shape a two-dimensional (2D) array of Bessel beams by a polarization-independent metasurface using Dammann gratings [35], while the resulting beams are at macro-scale rather than subwavelength dimensions. Additionally, the high-intensity zero-order spot greatly affects the uniformity of the array spot, the latter being only 42.15% even when the zero-order spot is excluded. Therefore, these beams are not applicable in realistic scenarios such as parallel laser fabrication or optical manipulation, which usually require high-resolution multiple optical beams with large intensity slopes and high uniformity of the beam intensity pattern.

In this endeavor, we utilized an ultra-thin all-dielectric metasurface to generate high-uniformity, high-resolution zeroth-order *J*_0_ and first-order *J*_1_ Bessel beam arrays based on the Dammann grating principle. By optimizing the Dammann grating and decreasing the size of each supercell of the grating, we realize a 4 × 4 array of Bessel beams with high uniformity intensity (52.40%) and subwavelength transverse dimension (570 nm, ∼0.9*λ*) for each beam with a large NA of 0.4. The uniformity of each beam intensity can be further increased to 86.5% which is twice larger than the value reported in the current literature [35], while the NA of the beam is 0.2 which is increased by 10 times. These Bessel beam arrays with high resolution and high uniformity have great potential for parallel processing techniques in the fields of optical manipulation [39], optical communication [40] and biomedical imaging [41, 42] at high penetration depths.

## 2 Results and discussion

### 2.1 Bessel-beam array generation principle

**Figure 1:**
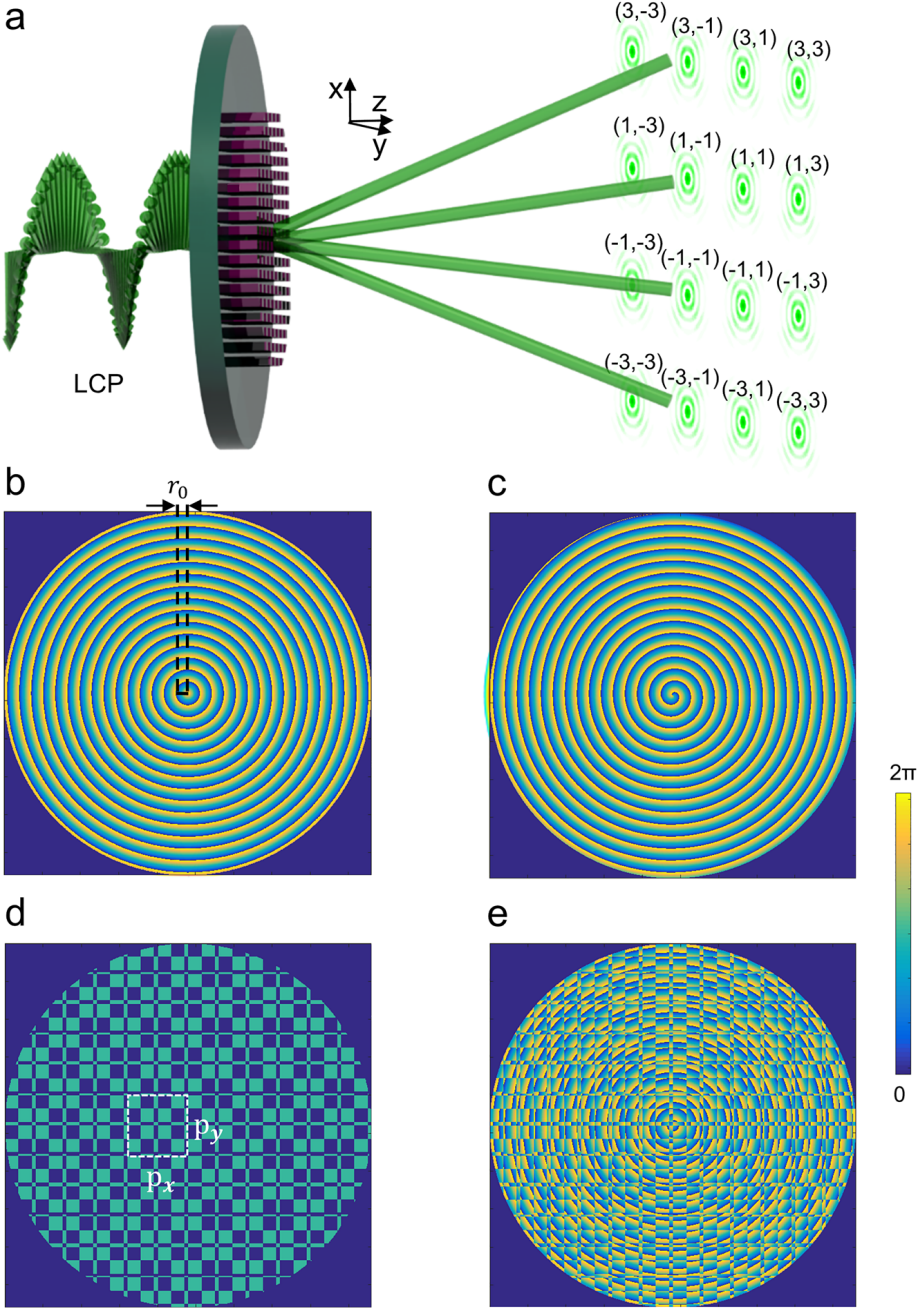
Generation principle of Bessel beam arrays through dielectric metasurface. (a) Schematic diagram of the generation process of the 4 × 4 array of Bessel beams. Phase distributions for the generation of (b) *J*_0_ Bessel beam, (c) *J*_1_ Bessel beam, (d) Dammann grating, (e) 4 × 4 *J*_0_ Bessel beam array (fusion of b and d).

A Bessel beam is a solution to the free space Helmholtz wave equation and the field distribution of a Bessel beam propagating in the non-diffractive direction of the *z*-axis is expressed as [21]:

(1) $$E\left(r,\varphi ,z\right)=A\cdot \text{exp}\left(ik_{z}z\right)\cdot J_{n}\left(k_{r}\right)$$

where *A* is the field amplitude, *k*_*z*_ and *k*_*r*_ are the longitudinal and transverse wave vectors, respectively, which fulfill the relation $\sqrt{k_{z}^{2}+k_{r}^{2}}=k$, in which ***k*** is the wave vector ($k=2\pi /\lambda_{\text{d}}$, where *λ*_d_ is the wavelength of the light). When left-handed circularly polarized (LCP) light is incident, this metasurface can generate a corresponding Bessel beam array through phase modulation. Figure 1a illustrates a schematic diagram of the generation process of the 4 × 4 array of Bessel beams based on our proposed dielectric metasurface.

As shown in Figure 1b, to obtain the zero-order Bessel beam, the phase profile of the metasurface is expressed as [21]:

(2) $$\begin{array}{l} \begin{array}{l} \varphi \left(x,y\right)=2\pi -\frac{2\pi }{\lambda_{\text{d}}}\cdot \sqrt{x^{2}+y^{2}}\cdot \text{NA} \end{array} \end{array}$$

where $\sqrt{x^{2}+y^{2}}=r$, *r* being the radial coordinate, and NA is the numerical aperture at the designated wavelength *λ*_d_. For a higher-order Bessel beam, a spiral phase is added to Eq. (2) (as shown in Figure 1c) and the phase profile is rewritten as:

(3) $$\begin{array}{l} \begin{array}{l} \varphi \left(x,y\right)=2\pi -\frac{2\pi }{\lambda_{\text{d}}}\cdot \sqrt{x^{2}+y^{2}}\cdot \text{NA}+n\Phi \end{array} \end{array}$$

where *Φ* = atan(*y*/*x*), *Φ* is the azimuthal angle and *n* is the topological charge number. It is worth noting that the phase distribution of a classical axicon is [43]:

(4) $$\begin{array}{l} \begin{array}{l} \varphi \left(x,y\right)=\frac{-2\pi \sqrt{x^{2}+y^{2}}}{r_{0}} \end{array} \end{array}$$

where the tunable parameter *r*_0_ is the distance of the period that covers a 2π phase as shown in Figure 1b. Using Eqs. (2) and (4), NA is calculated as NA = *λ*_d_/*r*_0_ for the case of a single Bessel beam.

In this study, we combined a meta-axicon with a Dammann grating [44] to generate a Bessel beam array. The Dammann grating is a type of diffraction grating designed to produce equal-intensity spots at different diffraction orders. Herein, a Dammann grating for generating an even number of spots is designed, which has better uniformity than a Bessel beam array with an odd number of spots [38, 45]. According to the diffraction principle for the Dammann grating, the diffraction angle *α* of each beam for a 2D grating is defined as [35]:

(5) $$\begin{array}{l} \begin{array}{l} \alpha =\text{asin}\left(\lambda \sqrt{{\left(\frac{i}{p_{x}}\right)}^{2}+{\left(\frac{j}{p_{y}}\right)}^{2}}\right) \end{array} \end{array}$$

where *p*_*x*_ and *p*_*y*_ are the grating period in the *x* and *y* directions (i.e., the size of the supercell shown in Figure 1d) and *i* and *j* are the diffraction orders in the *x* and *y* directions. Additionally, the distance between the spot of the (*i*, *j*)-th diffraction order and the optical axis is defined as:

(6) $$\begin{array}{l} \begin{array}{l} \Delta xy=\frac{D}{2\;\tan\left(\text{asin}\left(\text{NA}\right)\right)}\times \tan\left(\text{asin}\left(\lambda \sqrt{{\left(\frac{i}{p_{x}}\right)}^{2}+{\left(\frac{j}{p_{y}}\right)}^{2}}\right)\right) \end{array} \end{array}$$

where *D* is the aperture of the metasurface. From Eq. (6) it can be seen that Δ*xy* becomes smaller when NA is increased (i.e., a more focused beam) and *D* is decreased (i.e., a higher integration degree of the device). Therefore, the phases of two neighboring spots will interact more intensively, which deteriorates both the quality and the uniformity of the beam. To increase uniformity, it is likely to increase the distance of the two neighboring spots by decreasing the values of *p*_*x*_ and *p*_*y*_. In our design, we optimize and minimize the value of *p*_*x*_ and *p*_*y*_ down to 8 µm (*p*_*x*_ = *p*_*y*_ = 8 µm, *D* = 6*p*_*x*_ = 48 µm), which is not realizable by conventional SLMs. This is because even a single pixel size of the SLM (e.g., 18 µm) is larger than the size of the supercell of the metasurface (*p*_*x*_ = *p*_*y*_ = 8 µm). Therefore, the metasurface has a particular advantage in generating high-uniformity beam arrays compared with SLMs.

**Figure 2:**
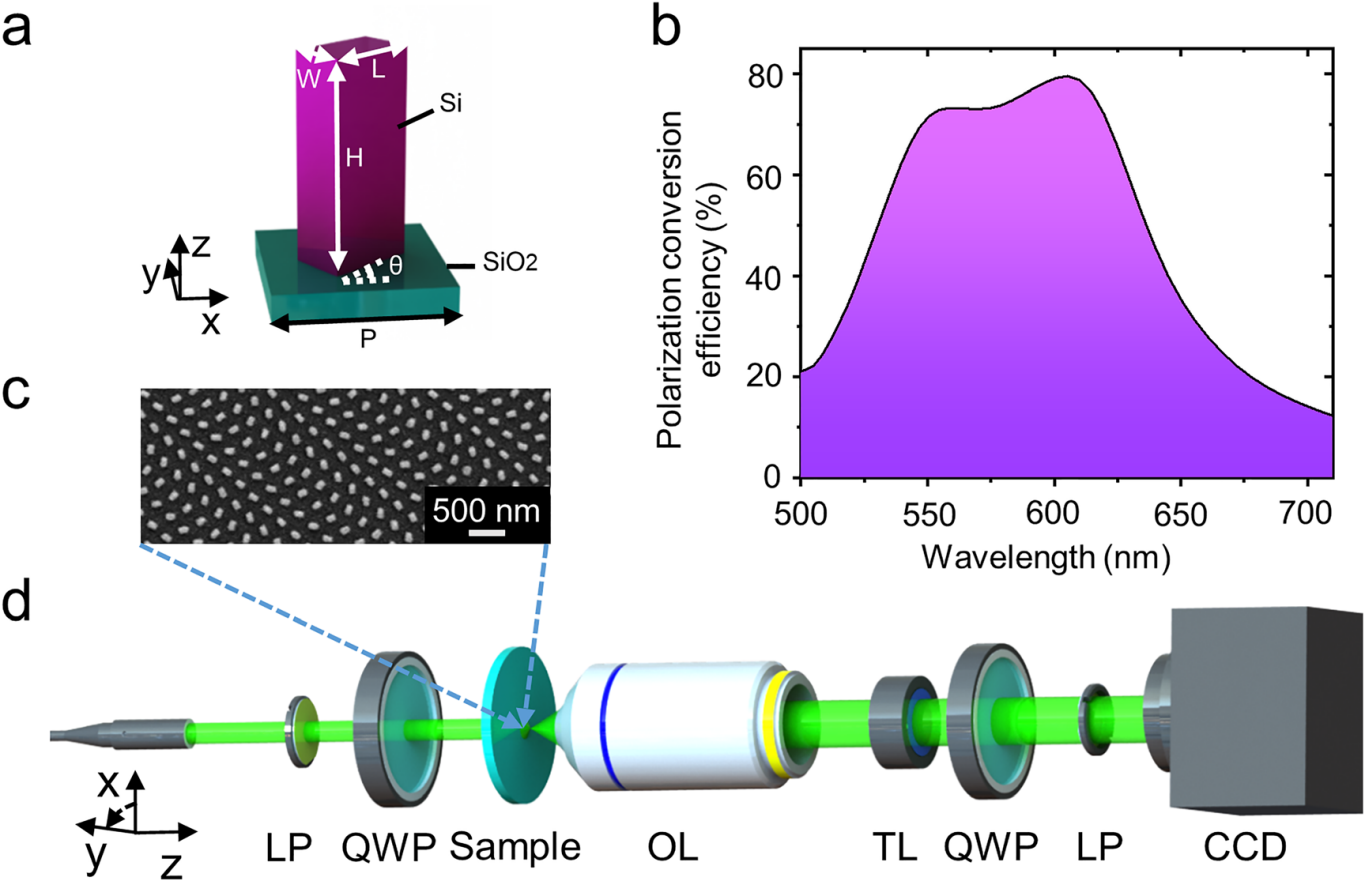
Metasurface design and experimental setup (a) Side view of the meta-atom: silicon nanopillar on a glass substrate indicating *L* = 130 nm as the length, *H* = 300 nm as the height, *W* = 80 nm as the width, and *P* = 250 nm as the period of the unit cell. (b) The polarization conversion efficiency at various wavelengths. (c) Top view of the SEM image of the fabricated metasurface sample. Scale bar: 500 nm. (d) Schematic of the Bessel beam array generator operating in the transmission mode and experimental setup for optical imaging of the generated pattern. The incident LCP light illuminates the generator along the positive *z*-axis. LP: linear polarizer; QWP: *λ*/4 quarter-wave plate; Sample: metasurface; OL: objective lens; TL: tube lens; CCD: charge-coupled device camera.

Following the above optimization process for the design of the Dammann grating, we design a metasurface for generating 1 × 4 and 4 × 4 arrays of Bessel beams. The one-dimensional (1D) Dammann grating is composed of 6 periodic supercells. Similar to the traditional Dammann grating [41] the phase of the designed supercell of the metasurface covers only 0 and π. Each supercell is composed of 32 nanopillars, as shown in Figure 2a. Its phase transition point is applied at the position of the 7th, 14th, 16th, 23rd, and 30th nanopillar, respectively, to preset the transition point close to the values 0.22057, 0.44563, 0.5, 0.72057, and 0.94563. Figure 1d shows the phase of a Dammann grating capable of generating a 4 × 4 array of Bessel beams. It is obtained by forming the product of two orthogonal 1D Dammann gratings. The phase pattern to generate a 4 × 4 array of *J*_0_ Bessel beams (Figure 1e), which is acquired by integrating the phases of the meta-axicon (Figure 1b) and the Dammann grating (Figure 1d), is:

(7) $$\begin{array}{l} \begin{array}{l} \varphi \left(x,y\right)=2\pi -\frac{2\pi }{\lambda_{\text{d}}}\cdot \sqrt{x^{2}+y^{2}}\cdot \text{NA}+\varphi_{\text{DG}}\left(x,y\right) \end{array} \end{array}$$

An additional vortex phase *n*Φ can be added to generate an array of Bessel vortex beams. Not only the aforementioned uniformity but also the maximum NA of Bessel beams are limited by conventional diffractive elements. As stated by the Nyquist sampling theorem, at least two discretization units of the phase plate cover a range of 2*π*, while the conventional diffractive elements with large pixel sizes lack the capability of phase manipulation in the subwavelength dimension, resulting in a low phase slope that limits the maximum NA. A Bessel beam array based on a liquid crystal display (LCD) that has a pixel size of 18 μm and the NA is 0.0027 has been demonstrated earlier [39]. However, at the designated wavelength of 488 nm, the maximum calculated NA according to the Nyquist sampling theorem is still limited to 0.014. In contrast, our designed metasurface can generate high-uniformity and high-resolution beam arrays compared with conventional SLMs.

### 2.2 Metasurface-based Bessel beam and beam array

The basic unit-cell of the metasurface is a silicon nanopillar with width (*W*), length (*L*), height (*H*), rotation angle (*θ*) and pitch (*P*) placed on a fused silica substrate as illustrated in Figure 2a. Following the Pancharatnam–Berry (PB) phase [46, 47], the rotation angle *θ* of the nanopillar is encoded by φ(x,y) in Eq. (7). When the circularly polarized light is converted into inverse circular polarized light, the transmitted inversed polarized light will have a geometric phase shift that is double the rotation angle *θ* of the nanorod. The lattice constant of the nanopillar is *P* = 250 nm in both *x* and *y* directions, which is adequate for generating Bessel beams at an incident light with a wavelength of 500–710 nm. Other design parameters, i.e., the length (*L*) and width (*W*) of the nanopillar, are optimized by finite-difference time-domain (FDTD) simulations executed with the commercial software FDTD Solutions (Lumerical Solutions Co. Ltd., Vancouver, BC, Canada). Periodic boundary conditions are applied in the *x* and *y* directions while perfectly matched layers are applied in the *z*-direction to achieve a minimum wave reflection. Adaptive meshes are used to conform to the shape of the geometry with a minimum mesh step of 0.25 nm. The calculated circular-polarization conversion efficiency for an optimized silicon nanopillar of *H* = 300 nm, *L* = 130 nm, *W* = 80 nm, and *P* = 250 nm is shown in Figure 2b. The optimized polarization conversion efficiency of 80% is obtained at *λ* = 608 nm (*n*_Silicon_ = 3.93 + 0.0233*i* at *λ* = 608 nm). Although silicon exhibits relatively high material losses in the range below 750 nm, we can still achieve this high efficiency due to the high real part of the refractive index of silicon in the visible region (*n*_Silicon_ = 4.08 + 0.0404*i* at *λ* = 550 nm and *n*_Silicon_ = 3.77 + 0.0114*i* at *λ* = 710 nm). After the geometry of the silicon nanopillar is designed, we fabricate the silicon metasurface by standard electron-beam lithography combined with a lift-off process (see fabrication details in the Experimental Section). A top-view scanning electron microscope (SEM) image of the fabricated metasurface is shown in Figure 2c. The experimental device for detecting the Bessel array spot is shown in Figure 2d (see the measurement details in the Experimental Section).

**Figure 3:**
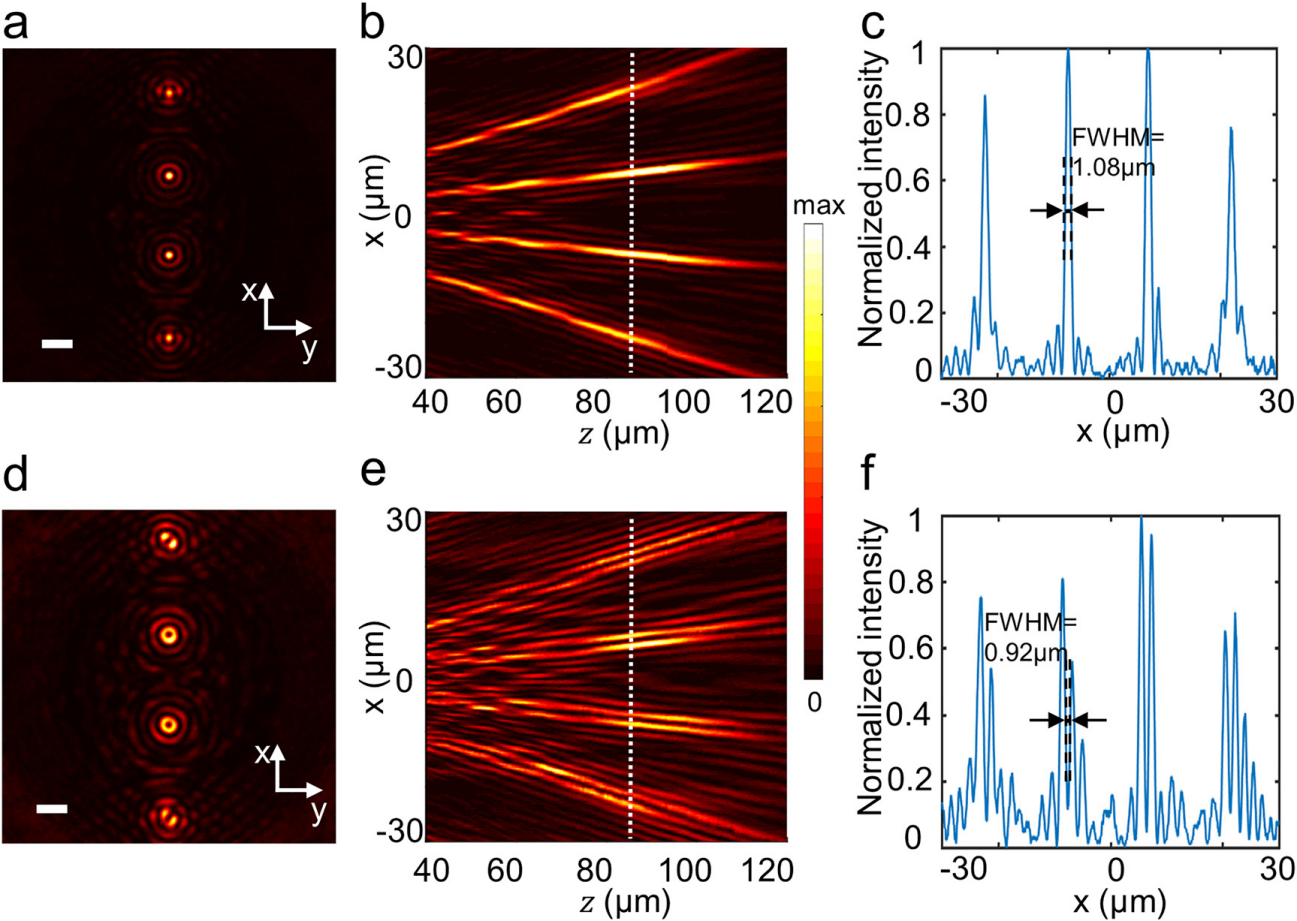
Light field distribution of Bessel beam array with NA = 0.2 at the designated wavelength *λ*_d_ = 630 nm. Measured transverse intensity distributions of 1 × 4 arrays of (a) *J*_0_ Bessel beams and (d) *J*_1_ Bessel beams, Scale bar = 10 µm. Measured longitudinal intensity distributions of 1 × 4 arrays of (b) *J*_0_ Bessel beams and (e) *J*_1_ Bessel beams. (c) and (f) are the extracted profiles from (b) and (e).

Figure 3 depicts the optical characterization of the 1 × 4 arrays of *J*_0_ and *J*_1_ Bessel beams with NA = 0.2 at the designated wavelength of *λ*_d_ = 630 nm. The phase distributions used to generate the *J*_0_ and *J*_1_ Bessel beam arrays are shown in Figure S1a and S1b, and the corresponding simulated transverse intensity profiles at the designated wavelength of *λ*_d_ = 630 nm are shown in Figure S1c and S1d. Figure S2 shows the influence of the size of the supercell of the Dammann grating on the uniformity of the 1 × 4 Bessel beam array. Figure 3a and d presents the measured transverse intensity profile of the 1 × 4 arrays of *J*_0_ and *J*_1_ Bessel beams. Meanwhile, the measured longitudinal intensity distributions of the above arrays shown in Figure 3b and e are sliced from the 3D field pattern, which is reconstructed from the transverse field distributions recorded at discrete locations along the beam propagation direction (see the measurement details in the Experimental Section). The theoretical depth of focus of a single *J*_0_ or *J*_1_ Bessel beam is *D*/2 tan(asin(NA)) = 117.6 μm (187*λ*) [21, 25], which is close to the experimental value of 115 μm. With reference to the position of the white dashed lines in Figure 3b and e, the extracted profiles are shown in Figure 3c and f, respectively. In the latter, it can be seen that the array pattern intensities are homogeneous among different spots without strong intensity from the zero-order light. The uniformities of intensity for the four spots, which is calculated as *Q* = 1 − (*I*_max_ − *I*_min_)/(*I*_max_ + *I*_min_) [36], are as high as 86.5% (*J*_0_ Bessel beam) and 70.13% (*J*_1_ Bessel beam). The *I*_max_ and *I*_min_ are the maximum and minimum central intensity of the four beams, respectively. It is important to emphasize that when the zero-order intensity of the generated Bessel beam array is relatively large [32], the uniformity of the intensity will be significantly affected. It can be observed from Figure 3c that the measured full width at half maximum (FWHM) of the *J*_0_ Bessel beam is 1.12 μm, which agrees well with the theoretical value, i.e., ${\text{FWHM}}_{J_{0}}=2.25/k_{r}=0.358r_{0}=1.13\text{ μm}$ (where *k*_r_ = 1.99 μm^−1^, *r*_0_ = 3.15 μm) [21, 25]. Figure 3f depicts that the measured FWHM of the *J*_1_ Bessel beam is 0.92 μm, which is quite close to the theoretical value, i.e., ${\text{FWHM}}_{J_{1}}=1.832/k_{r}=0.292r_{0}=0.935\text{ μm}$ (where *k*_r_ = 1.99 μm^−1^, *r*_0_ = 3.15 μm) [21, 25].

**Figure 4:**
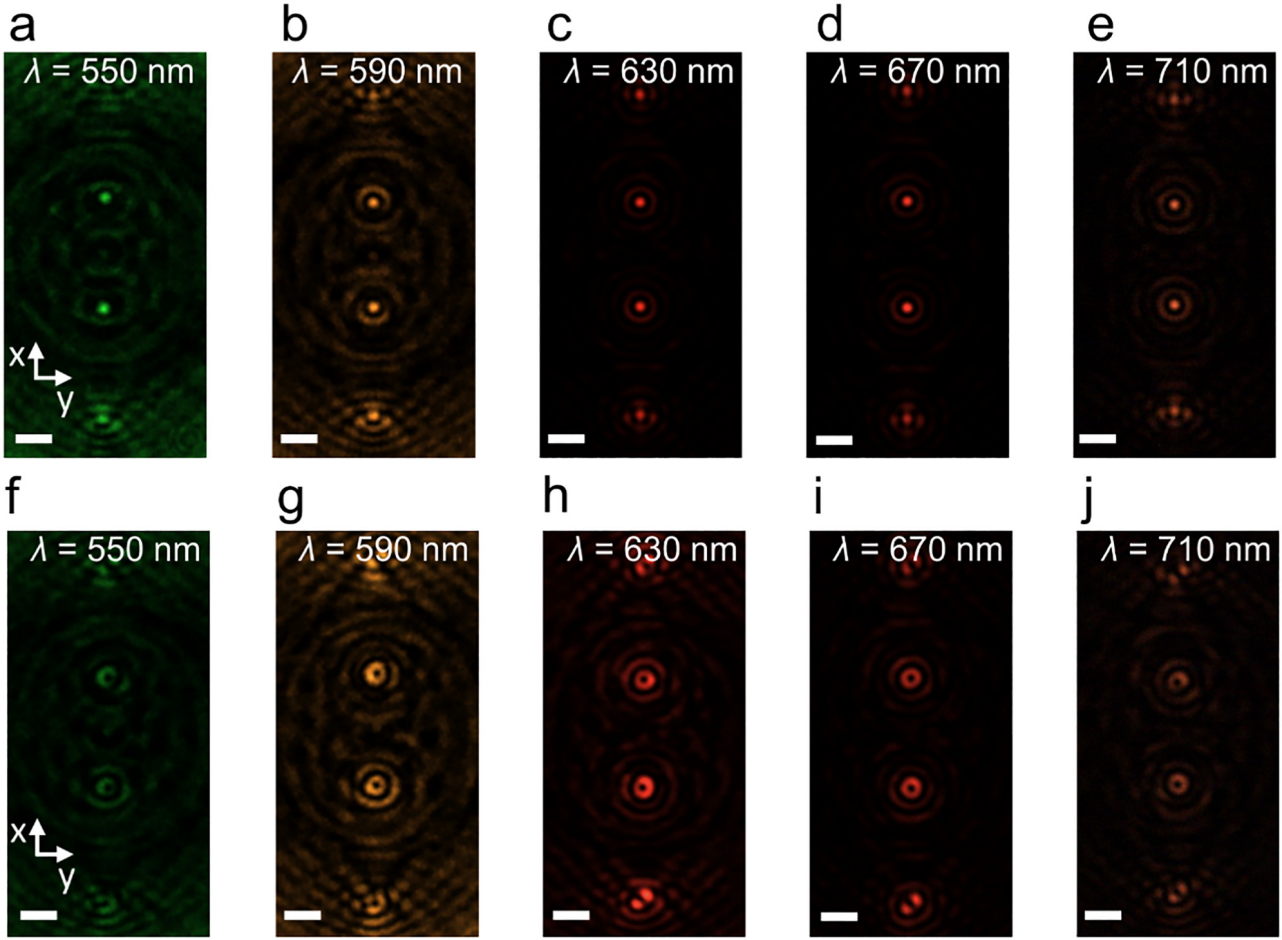
Measured transverse intensity distributions of *J*_0_ (a–e) and *J*_1_ (f–j) Bessel beam arrays at the vertical location of *z* = 90 μm at wavelengths *λ* = 550, 590, 630, 670, and 710 nm, respectively.

**Figure 5:**
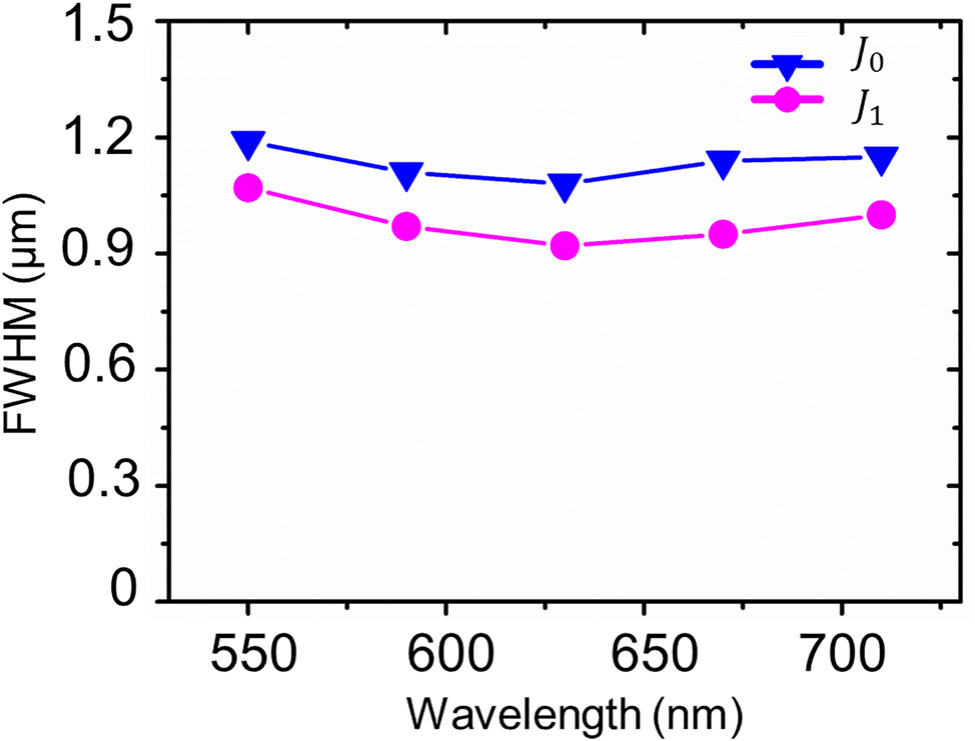
FWHMs of *J*_0_ (blue solid line) and *J*_1_ (pink solid line) Bessel beams at various wavelengths.

To demonstrate the broadband characteristics of the proposed device due to the PB phase, *J*_0_ and *J*_1_ Bessel beam arrays were experimentally measured at various wavelengths (*λ* = 550, 590, 630, 670, and 710 nm) and the results are shown in Figure 4a–e and f–j at *z* = 90 μm. It is worth noting that, likewise to the meta-axicons in another study [37], the FWHMs of *J*_0_ and *J*_1_ Bessel beam arrays remain constant for different wavelengths, which follows the fact that the aforementioned theoretical formulas for the FWHMs rely only on a fixed value of *r*_0_ [25]. Figure 5 summarizes the measured FWHMs of *J*_0_ and *J*_1_ Bessel beams extracted from Figure 4 at various wavelengths. Their average values are 1.13 µm varying between 1.08 and 1.19 µm for *J*_0_ Bessel beams, and 0.98 µm varying between 0.92 and 1.07 µm for *J*_1_ Bessel beams, which match the wavelength-independent behavior of the theoretical values.

**Figure 6:**
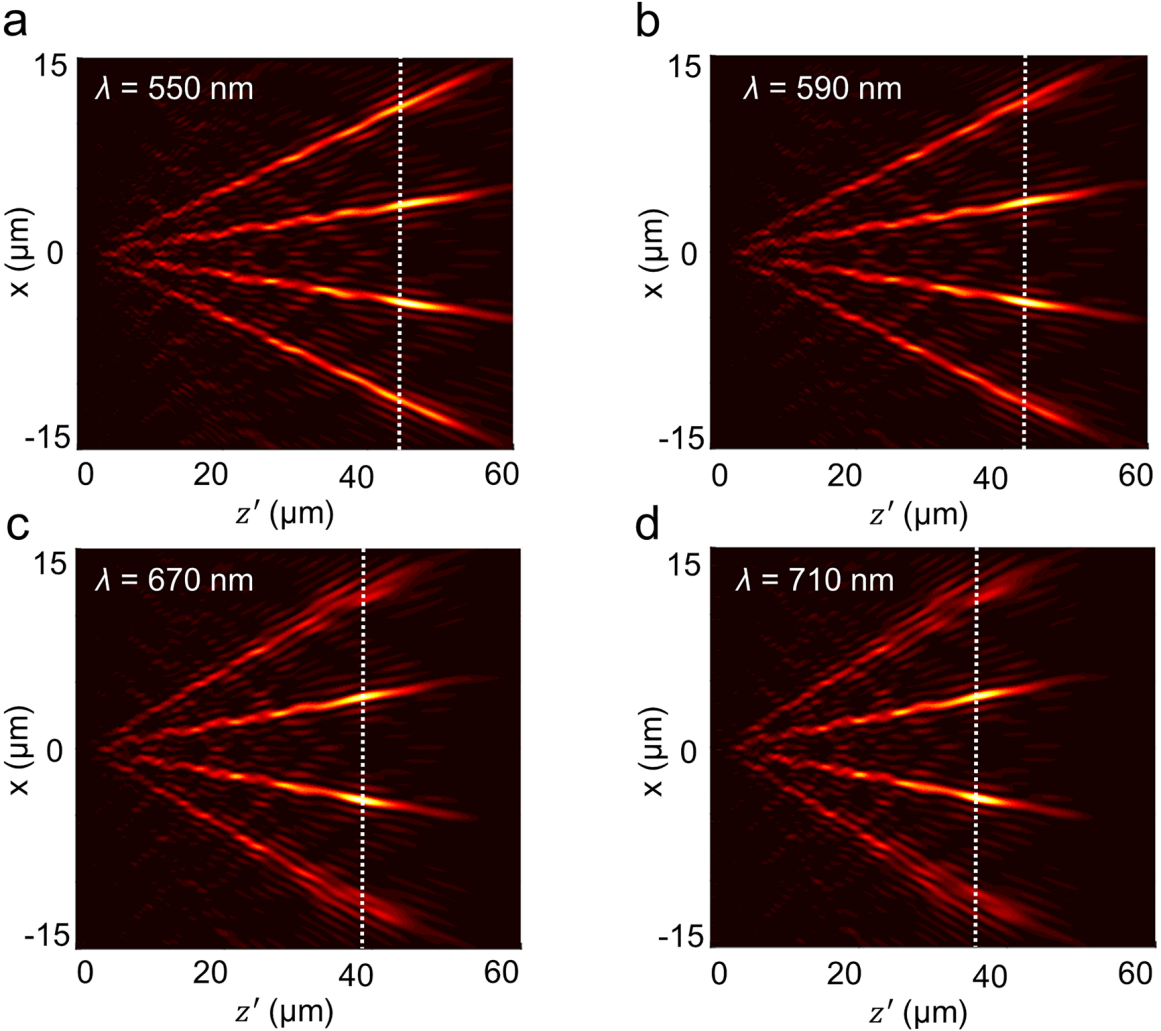
FDTD simulations of a 1 × 4 array of Bessel beams with NA = 0.4 (*λ* = 630 nm,) at various wavelengths *λ* = 550 nm, 590 nm, 630 nm, 670 nm, and 710 nm.

While keeping other design parameters unaltered, we increased the NA of the Bessel beams to 0.4 to obtain a Bessel beam array with sub-wavelength transverse dimensions for the single beam. The longitudinal optical field patterns at *λ* = 550, 590, 670 and 710 nm in the *x*–*z*ʹ plane are displayed in Figure 6. It can be observed that the field intensity of the first diffraction order is larger than that of the second diffraction order. Furthermore, Figure 6a–d depict that as the incident wavelength decreases from 710 to 550 nm, there is a gradual increase in the non-diffractive propagation distance. Meanwhile, the beam pattern at *λ* = 550 nm becomes even more homogeneous with uniformity of 70.99%, which is larger than that of the other arrays, which have uniformity values of 57.22% at *λ* = 590 nm, 46.4% at *λ* = 670 nm and 43.47% at *λ* = 710 nm. The simulated non-diffractive propagation distances of the Bessel beam in the array at *λ* = 710, 670, 590 and 550 nm are 45, 50, 56 and 60 μm, respectively, which are close to the theoretical values of a single beam of 48, 51, 59 and 64 μm. It should be noted that once *r*_0_ is specified, its non-diffractive propagation distance is proportionate to the size of the metasurface at a certain wavelength. The non-diffractive propagation distance of the beam can be appropriately increased by reducing the NA of the beam or expanding the size of the device. Additionally, the deflection angle of the array beam can be enlarged by increasing the size of the supercell of the Dammann grating.

**Figure 7:**
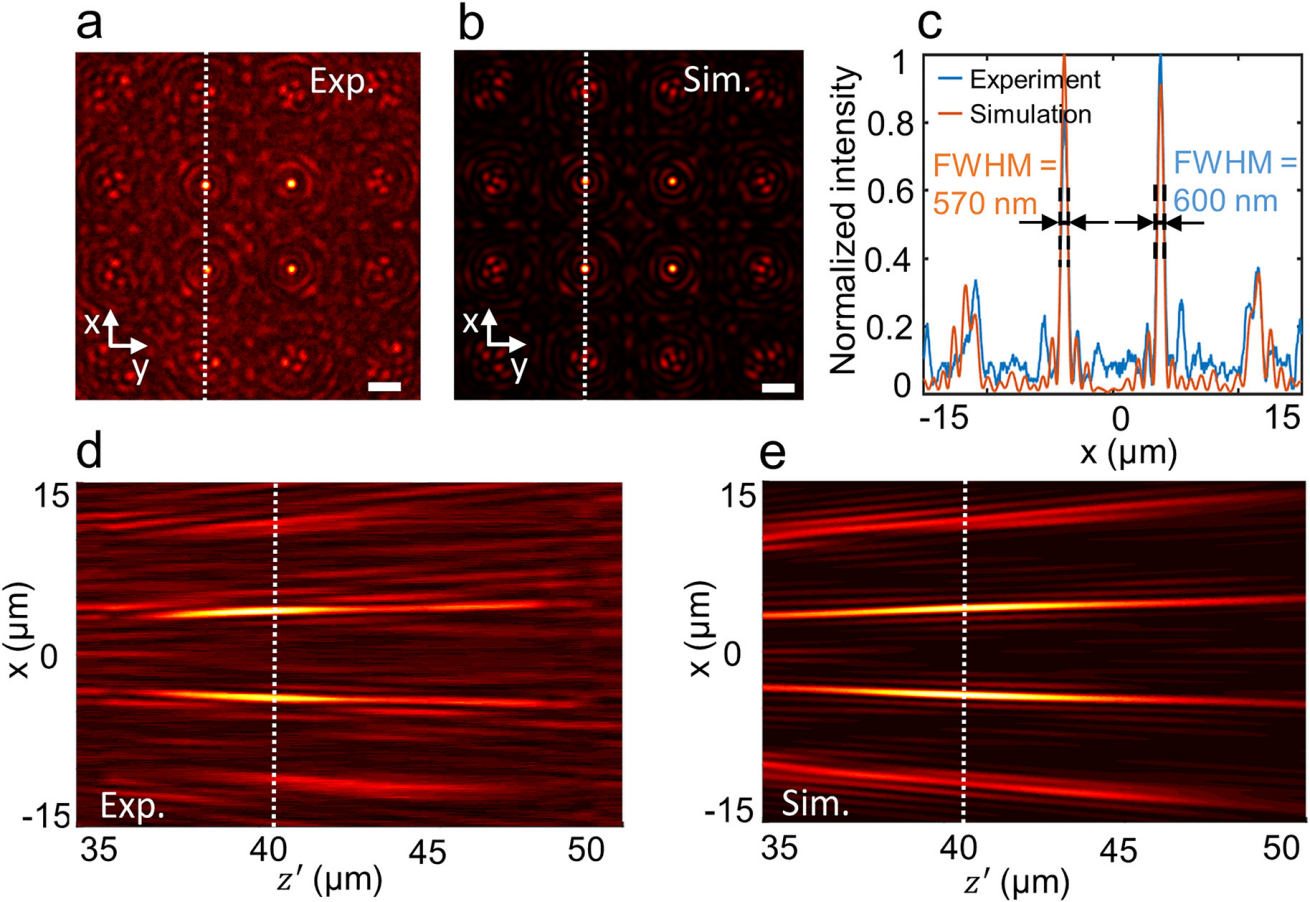
Simulation and experimental results of Bessel beam arrays (a) Experimental and (b) simulated transverse field distributions of the 4 × 4 array of Bessel beams with NA = 0.4 at the designated wavelength *λ*_d_ = 630 nm in the *x*–*y* plane. Scale bar = 5 µm. (c) The orange and blue profiles represent the cross-section distributions at the position of the white dashed line in (a) and (b). (d) Experimental and (e) simulated longitudinal field distributions of the beam pattern along the propagation direction.

Apart from the demonstration of the 1D 1 × 4 arrays of Bessel beams, we additionally validate the performance of the proposed device in generating 2D Bessel beam arrays. Figures 7a and b depict the experimental and simulated transverse field distributions of 2D 4 × 4 arrays of Bessel beams with NA = 0.4 at the designated wavelength of *λ*_d_ = 630 nm. The orange and blue profiles shown in Figure 7c refer to the simulated and experimental cross-section profiles extracted at the position of the white dashed lines in Figure 7a and b. It can be seen in Figure 7c that the experimental value of the FWHM is 600 nm, whereas its simulated and theoretical values are 570 and 564 nm, respectively. The experimental and simulated longitudinal field distributions of the beam patterns along the propagation direction are shown in Figure 7d and e, respectively. The measured non-diffractive propagation length is 48 µm, which matches well with the theoretical value of 55 µm [21, 25]. Therefore, the obtained experimental results show good agreement with both simulations and theoretical analysis. The uniformities of the arrays in Figure 7d and e are 52.40 and 54.01%, respectively.

It is worth noting that the high diffraction ordered Bessel beams in the array are distorted as shown in Figure 7a. Even though the diffraction angle (18.95°) of the (±3, ±1)-th diffraction ordered beam in Figure 7a is close to the value (18.36°) of the (±3, 0)-th diffraction ordered beam in Figure 3a, the quality of the beam in Figure 7a is significantly lower than that in Figure 3a. It shows that the Bessel beams with NA of 0.4 have more distortion than that with NA of 0.2. In addition, the energy passed through the metasurface is distributed over the 4 × 4 beams, leading to a relatively low signal intensity for each beam. And in the experiment, the polarization efficiency is 55% which means nearly a half of the energy of the incident beam needs to be filter out. The above combined factors cause the relatively low signal to noise ratio of the beams in Figure 7a and b. To reduce the distortion of the high diffraction ordered Bessel beam in Figure 7, we increased the size of the supercell of the Dammann grating *p*_*x*_ (*p*_*y*_) to 9.6 μm as shown in Figure S3a. The simulated light field distribution in the *x*–*y* plane is shown in Figure S3c. It shows that the quality of the Bessel beam array is significantly improved and the uniformity of the (*i*, 1)-th (*i* = ±1, ±3) diffraction ordered beam achieves 92.01%. Next, we increase the size of the supercell of the Dammann grating *p*_*x*_ (*p*_*y*_) further to 12 μm as shown in Figure S3b. However, the quality of the Bessel beam array begins to decrease as shown in Figure S3d. The uniformity of the (*i*, 1)-th (*i* = ±1, ±3) diffraction ordered beam reduces to 77.86%. It might be because that the side lobes of the adjacent Bessel beams become more overlapping and affect each other. The conversion efficiencies of Bessel beams with different diffraction orders in Figure S1c, S1d and S3c are shown in Tables S1–S3.

Table 1 summarizes the values of uniformity of the arrays with different beam parameters. By utilizing our designed metasurface, we find that contrary to the corresponding values found in another study [35], the uniformity of the arrays in Figure 3b is enhanced significantly, i.e., almost double, while the NA of the beams in Figure 3b is increased by more than 10 times. Furthermore, when the NA of the beams in Figure 7d and e is 0.4 which is increased by more than 20 times, the uniformities of the arrays are still better than the corresponding values [35]. As a result, our designed metasurface with optimized Dammann grating produces Bessel beam arrays with higher resolution and better uniformity than the conventional SLMs and other metasurface designs.

**Table 1:** Summary of the uniformity values of the arrays with different beam parameters.

Figure	Beam type	NA of beams in the array	Uniformity %
3b	1 × 4 array of *J*_0_ Bessel beams	0.2	86.50 at *λ* = 630 nm
3e	1 × 4 array of *J*_1_ Bessel beams	0.2	70.13 at *λ* = 630 nm
6a	1 × 4 array of *J*_0_ Bessel beams	0.35	70.99 at *λ* = 550 nm
6b		0.37	57.22 at *λ* = 590 nm
6c		0.43	46.40 at *λ* = 670 nm
6d		0.45	43.47 at *λ* = 710 nm
7d	4 × 4 array of *J*_0_ Bessel beams	0.4	52.40 at *λ* = 630 nm
7e	4 × 4 array of *J*_0_ Bessel beams	0.4	54.01 at *λ* = 630 nm
5a in ref. [35]	5 × 5 array of *J*_0_ Bessel beams	0.0195 (NA = *λ*_d_/*r*_0_) *r*_0_ = 40 µm, *λ*_d_ = 780 nm	42.15 at *λ* = 780 nm

## 3 Conclusions

In this research, a novel method to design high-uniformity and high-resolution *J*_0_ and *J*_1_ Bessel beam arrays by means of all-dielectric silicon metasurfaces is introduced. Subsequently, the method is demonstrated through detailed experiments and the results agree well with both simulations and theoretical analysis. In more detail, the FWHM of the spots of the 4 × 4 array of *J*_0_ Bessel beams (NA = 0.4) is as small as 570 nm (0.9*λ*) at the designated wavelength *λ*_d_ = 630 nm, while the uniformity of each beam intensity in the array is maintained as high as 52.40%. Furthermore, the uniformity of the 1 × 4 *J*_0_ Bessel beam array with NA = 0.2 reaches as high as 86.5%. Alike to a single Bessel beam, the FWHMs of the Bessel beam arrays are wavelength-independent. Compared with the former methods of generating and controlling Bessel beam arrays, our design strategy provides superior high-uniformity and high-resolution characteristics by optimizing the supercell of the Dammann grating. We firmly believe that our proposed strategy of generating Bessel beam arrays can be utilized to provide compact and highly integrated platforms for generating various practical types of non-diffractive beam arrays.

## 4 Experimental section

*Sample fabrication*: E-beam lithography and lift-off processes were used to realize the metasurfaces. Our design utilizes silicon as the optical material because it ensures relatively high polarization conversion efficiency with a rigorous optimization of the geometric parameters of the nanorod. Initially, a 300-nm-thick uniform amorphous silicon layer was deposited by a sputtering machine. Then, a layer of resist ZEP520 with a thickness of 350 nm was spin-coated on the wafer. Afterward, the hot plate was baked at 180 °C for 1 min. E-beam lithography was operated using an electron beam system (JEOL JBX6300fs) with an acceleration voltage of 100 keV. The sample was then developed in amyl acetate solution at room temperature for 65 s. An inductively coupled plasma–reactive ion etching (ICP-RIE) machine was used for silicon etching.

*Optical characterization*: The experimental optical setup for the measurement of field distributions of the generated Bessel beam array is displayed in Figure 2d. The collimated beam from the NKT super-continuum light source (NKT Photonics SuperK EXTREME EXR-15) was manipulated to an LCP beam by using a polarizer (Thorlabs LPVIS100) and a quarter-wave plate (Thorlabs AQWP05M-600). The incident light illuminated the metasurface perpendicularly. Initially, the transmitted fields of the generated Bessel beam array from the metasurface were magnified by an Olympus objective (NA = 0.65, 40×) for Figures 3a, b, d, e and 4a–j or a Leica objective (NA = 0.85, 100×) for Figure 7a and d and a tube lens (*f* = 180 mm), and subsequently, these fields were imaged by a CCD camera (DH-HV3151UC). Another quarter-wave plate and a polarizer were positioned between the tube lens and the CCD camera to filter out the transmitted inverse circularly polarized light, i.e., RCP light. The objective lens and the tube lens were fixed on a high-speed motorized *xy* scanning stage (Thorlabs MLS230-1), which moved along the longitudinal direction with a step size of 1 μm so that the magnified transverse field slices could be recorded at these discrete longitudinal positions. A home-made LabVIEW program was used to synchronize the time of the CCD captured image and the movement of the motor stage. Finally, these recorded 2D *xy* field distribution slices on each discrete longitudinal position along the incident beam propagation direction were superimposed and reconstructed to 3D field patterns, from which the longitudinal *xz* field profiles of the 2D Bessel beam array were extracted as shown in Figures 3b, e and 7d.

## Supplementary Material

Supplementary Material
