# Supplementary material for: Generation of high-uniformity and high-resolution Bessel beam arrays through all-dielectric metasurfaces
Source: Nanophotonics. 2022 Jan 11;11(5):967–77. doi: 10.1515/nanoph-2021-0603 (PMC11501921; doi:10.1515/nanoph-2021-0603)
Supplement: Supplementary file 1 — Supplementary Material [file j_nanoph-2021-0603_suppl.docx]

Supporting information for

**Generation of High-uniformity and High-resolution Bessel Beam Arrays through All-dielectric Metasurfaces**

Lei Chen^1^, Saima Kanwal^1^, Binbin Yu^2,3^, Jijun Feng^1^, Chunxian Tao^1^, Jing Wen^1^* and Dawei Zhang^1^

^1^Engineering Research Center of Optical Instrument and Systems, Ministry of Education and Shanghai Key Lab of Modern Optical System, University of Shanghai for Science and Technology, No. 516 Jun Gong Road, Shanghai. 200093, China

^2^University of Chinese Academy of Sciences, Wenzhou, Zhejiang 325000, China

^3^Oujiang Laboratory, Wenzhou, Zhejiang 325000, China

*Corresponding Author: jwen@usst.edu.cn

**Simulation results of Bessel beam arrays**

**Ⅰ. Optimize the size of the super cell of the Dammann grating**

Figure S1 shows the 1 × 4 *J*_0_ and *J*_1_ Bessel beam arrays by optimizing the size of the supercell of the Dammann grating. The total size of the device is 48 µm, and the generated Bessel beam array has NA = 0.2. Figures S1a and S1b respectively show the phases of the Dammann grating composed of 1 × 6 super cells for generating the 1 × 4 *J*_0_ and *J*_1_ Bessel beam arrays. Figures S1c and S1d respectively show the electric field distributions of the corresponding *J*_0_ and *J*_1_ Bessel beam arrays in the *x*-*y* plane.

Figure S2 shows the influence of the size of the supercell of the Dammann grating on the uniformity of the 1 × 4 Bessel beam array. The total size of the device is 48 µm, and the generated Bessel beam array has NA = 0.2 at the designated wavelength *λ*_d_ = 630 nm. Figures S2a and S2b show the phases of the Dammann grating composed of 1 × 11 and 1 × 4 super cells for generating the 1 × 4 *J*_0_ and *J*_1_ Bessel beam arrays. Figures S2c and S2d respectively show the electric field distributions of the corresponding *J*_0_ Bessel beam arrays in the *x*-*y* plane.

As shown in Figure S2a, we use the parameters of the metasurface for generating the Bessel beam in the manuscript (NA = 0.2, D = 48 μm) and reduce the size of the supercell of the Dammann grating *p_x_* (*p_y_*) to 4.4 μm (i.e., there are 11 super cells in the Dammann grating). The diffraction angle of the ± 3rd order beam α is about 25°. Since the ±3rd order Bessel beam deviates from the optical axis by a large angle, the electric field distribution of the 3rd order Bessel beam is blurred as shown in Figure S2c. Then we increase the size of the supercell of the Dammann grating *p_x_* (*p_y_*) to 12 μm (that is, there are 4 supercells in total) as shown in Figure S2b. The diffraction angle of the ± 3rd order beam α decreases to 9.06°. Due to the small deviation of the ±3rd order Bessel beam from the optical axis, the quality of the ±3rd order Bessel beam has been significantly improved as shown in Figure S2d. Concerning the ±1st order Bessel beam, the diffraction angle in Figure S2d is 3.01° which is smaller than the diffraction angle (8.3°) in Figure S2c. Thus, the distance between the ±1st order Bessel beams in Figure S2d is smaller than that in Figure S2c. In Figure S2d, the side lobes of the neighboring ±1st order Bessel beams overlap more than Figure S2c. This makes the quality of ±1st order Bessel beam in Figure S2d significantly worse than that in Figure S2c.

In order to keep the diffraction angle not too large from the optical axis, the size of the supercell of the Dammann grating *p_x_* (*p_y_*) cannot be infinitely small. And at the same time, the size of the supercell of the Dammann grating *p_x_* (*p_y_*) can not be too large, because the quality of ±1st order Bessel beam will be influenced. As shown in Figure S1, we finally optimize the size of the supercell of the Dammann grating *p_x_* (*p_y_*) to be 8 microns.

Figure S3 shows the influence of the size of the supercell of the Dammann grating on the uniformity of the 4 × 4 Bessel beam array. The total size of the device is 48 µm, and the generated Bessel beam array has NA = 0.4 at the designated wavelength *λ*_d_ = 630 nm. Figures S3a and S3b show the phases of the Dammann grating composed of 5 × 5 and 4 × 4 super cells for generating the 4 × 4 *J*_0_ Bessel beam arrays. Figures S3c and S3d respectively show the electric field distributions of the corresponding *J*_0_ Bessel beam arrays in the *x*-*y* plane. The uniformity of the *J*_0_ Bessel beam array in Figure S3c is significantly higher than that in Figure S3d.


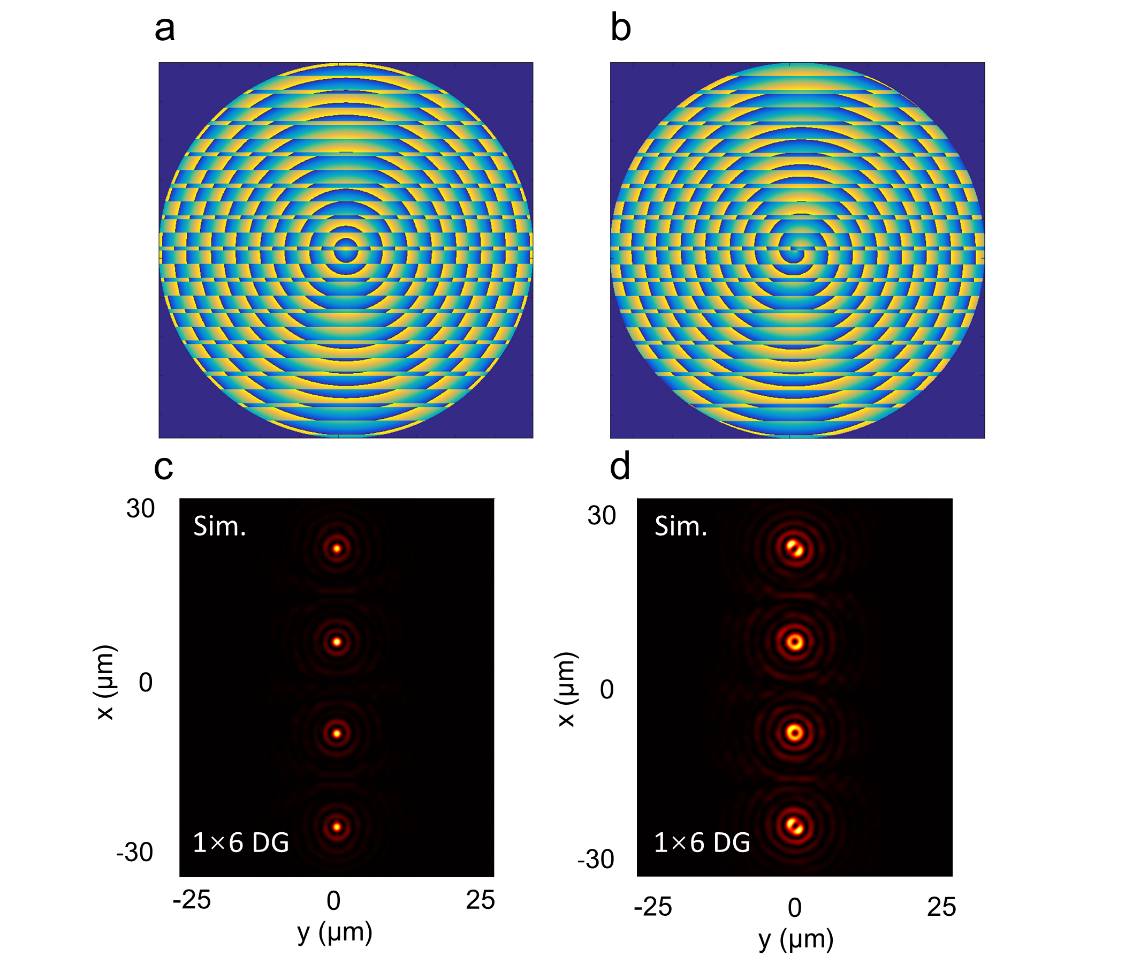


**Figure S1.** Phases of the Dammann grating composed of 1 × 6 super cells for generating 1 × 4 *J*_0_  (a) and *J*_1_ (b) Bessel beam arrays (NA = 0.2 at designated wavelength *λ*_d_ = 630 nm). DG represents a supercell of the Dammann grating. (c–d) Simulated electric field distributions of the corresponding *J*_0_ and *J*_1_ Bessel beam arrays in the *x*-*y* plane.


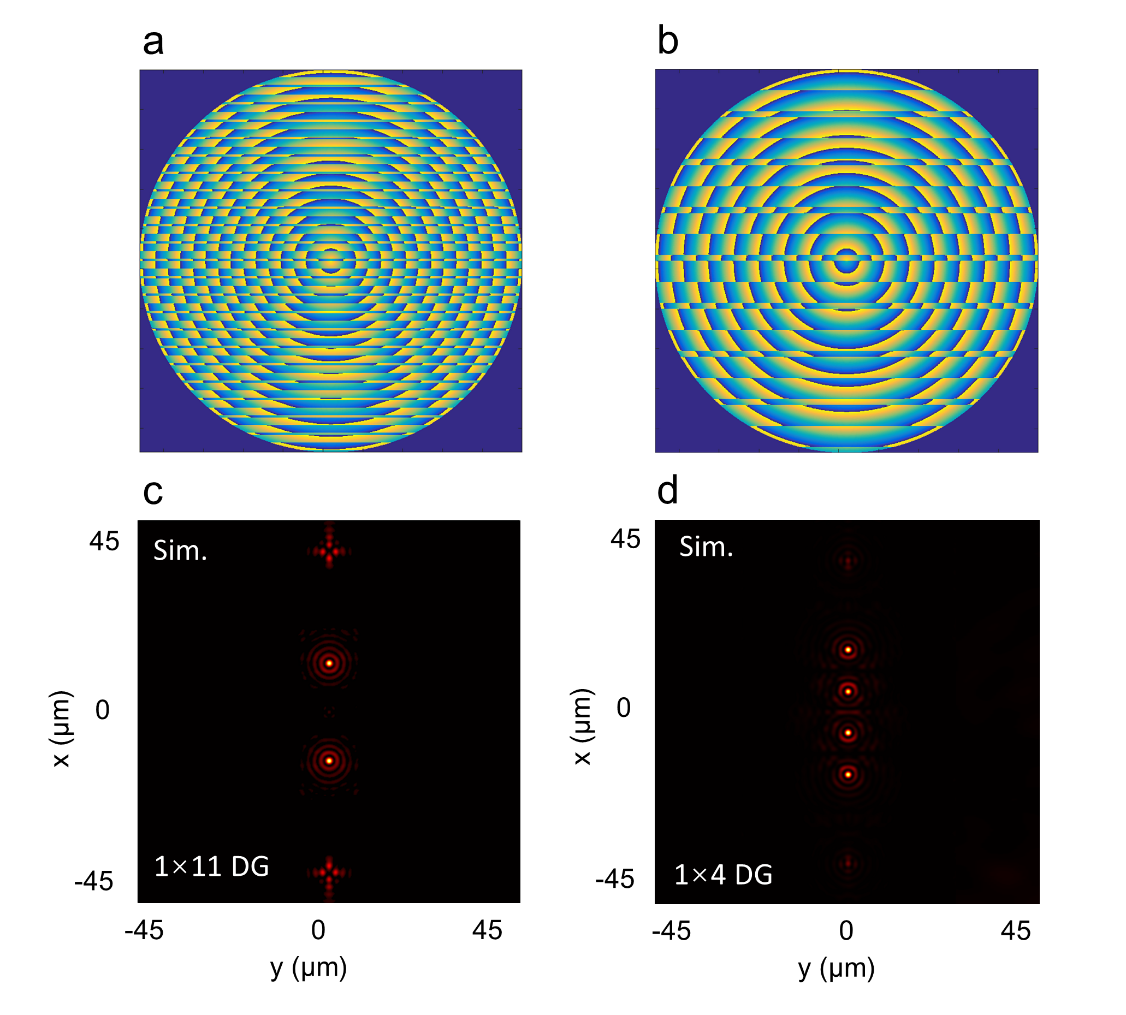


**Figure S2.** (a–b) Phases of the Dammann grating composed of 1 × 11 and 1 × 4 super cells for generating 1 × 4 *J*_0_  Bessel beam arrays (NA = 0.2 at designated wavelength *λ*_d_ = 630 nm). DG represents a supercell of the Dammann grating. (c–d) Simulated electric field distributions of the corresponding *J*_0_ Bessel beam arrays in the *x*-*y* plane.


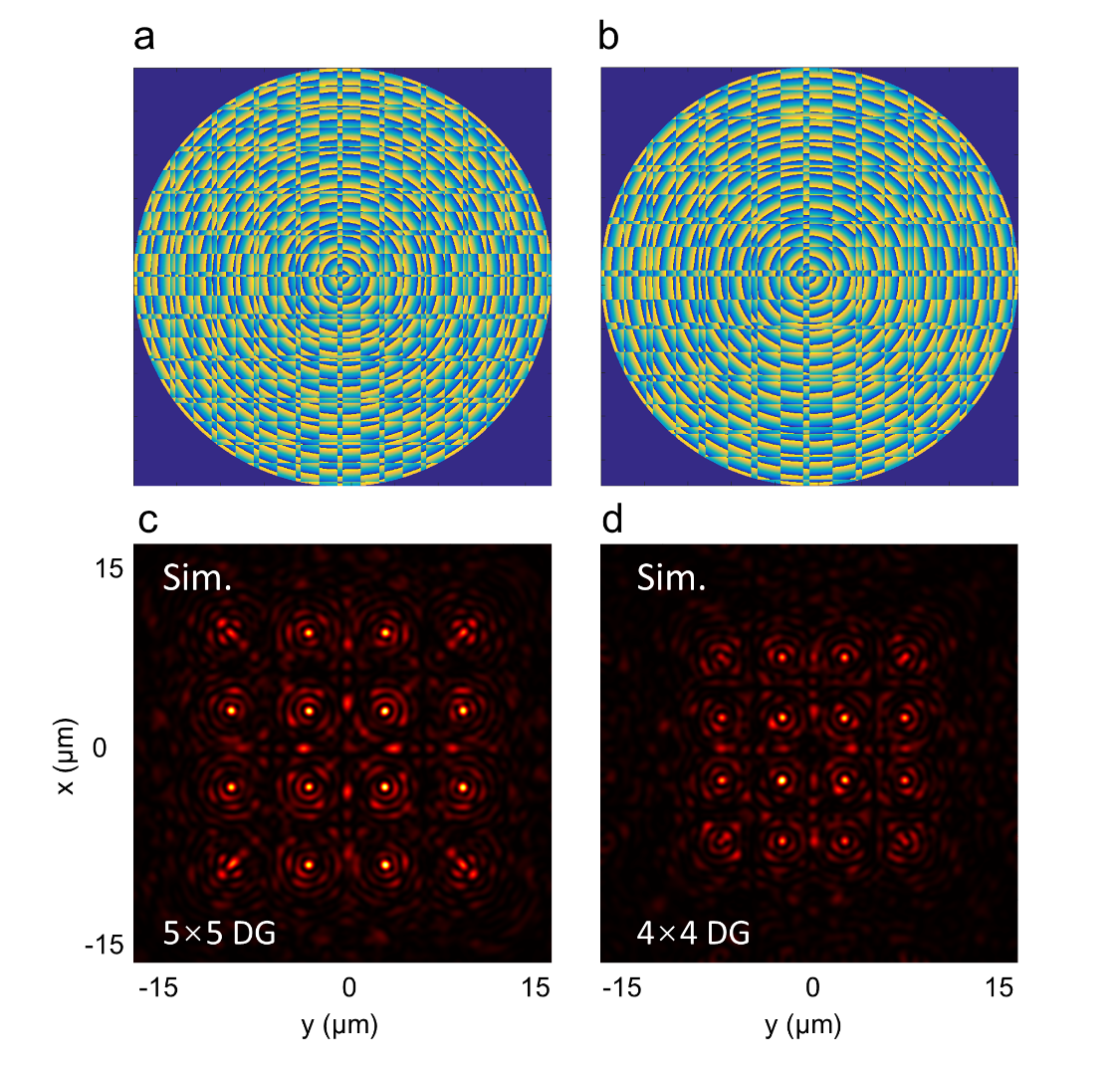


**Figure S3.** (a–b) Phases of the Dammann grating composed of 5 × 5 and 4 × 4 super cells for generating the 4 × 4 *J*_0_ Bessel beam arrays (NA = 0.4 at designated wavelength *λ*_d_ = 630 nm). DG represents a supercell of the Dammann grating. (c–d) Simulated electric field distributions of the corresponding 4 × 4 *J*_0_ Bessel beam arrays in the *x*-*y* plane.

**Ⅱ. Diffraction efficiency of Bessel beam arrays**

Tables S1–S3 show the simulated conversion efficiencies of Bessel beams with different diffraction orders in Figures S1c, S1d and Figure S3c. Their total conversion efficiencies are 33.25%, 35.25% and 20.23%, respectively.

Table S1: The efficiencies of Bessel beams with different diffraction orders in Figure S1c

i

| j | -3 | -1 | 1 | 3 |
| --- | --- | --- | --- | --- |
| 0 | 8.34% | 8.26% | 8.42% | 8.23% |

Table S**2**: The efficiencies of Bessel beams with different diffraction orders in Figure S1d

i

| j | -3 | -1 | 1 | 3 |
| --- | --- | --- | --- | --- |
| 0 | 8.94% | 8.97% | 8.94% | 8.40% |

Table S**3**: The efficiencies of Bessel beams with different diffraction orders in Figure S3c

i

| j | -3 | -1 | 1 | 1 |
| --- | --- | --- | --- | --- |
| 3 | 1.06% | 1.24% | 1.30% | 1.04% |
| 1 | 1.30% | 1.45% | 1.52% | 1.27% |
| -1 | 1.27% | 1.41% | 1.41% | 1.26% |
| -3 | 1.13% | 1.20% | 1.30% | 1.07% |
